# Altered digit tip blastema differentiation and bone regeneration in skeletally mature Ts65Dn Down syndrome mice

**DOI:** 10.1016/j.bone.2025.117648

**Published:** 2025-09-16

**Authors:** Sarah M. Wolff, Ling Yu, Mingquan Yan, Regina Brunauer, Margarita Rodriguez, David I. Garcia, Ashima Jain, Dimas R. Kusuma, Kirby M. Sherman, Cole B. Dahlstrom, Dana Gaddy, Larry J. Suva, Lindsay A. Dawson

**Affiliations:** a Department of Veterinary Physiology and Pharmacology, College of Veterinary Medicine and Biomedical Sciences, Texas A&M University, College Station, TX, 77843, United States of America; b LBG Ludwig Boltzmann Institute for Traumatology, The Research Center in Cooperation with AUVA, 1200, Vienna, Austria; c Austrian Cluster for Tissue Regeneration, 1200, Vienna, Austria; d Department of Veterinary Integrative Biosciences, College of Veterinary Medicine and Biomedical Sciences, Texas A&M University, College Station, TX, 77843, United States of America

**Keywords:** Digit regeneration, Blastema, Ts65Dn, Down syndrome, Intramembranous ossification, P3, Trisomy 21

## Abstract

Down syndrome (DS), the result of Trisomy 21 (T21), is associated with accelerated aging and impacts many organ systems across the lifespan, including the musculoskeletal system. Skeletal deficits such as low bone mineral density predispose the T21 population to skeletal injuries, especially as they age, and likely reduce their capacity to repair bone. Previous studies have demonstrated impaired secondary fracture healing in 4-month-old DS mice and diminished bone regeneration in young (2-months-old) DS mice. To investigate how bone regeneration is further impacted in skeletally mature (6-months-old) mice, terminal phalanx (P3) digit tip amputations were performed in a murine model of DS, Ts65Dn mice. The P3 regeneration cascade is characterized by an initial phase of bone degradation followed by intramembranous ossification to restore the amputated bone. These studies demonstrate that the bone regeneration anomalies observed in young Ts65Dn mice are exacerbated in skeletally mature mice, characterized by a complex dysregulation of bone resorption and formation genes. Collectively, skeletally mature Ts65Dn mice show fundamental *in vivo* deficits in progenitor cell differentiation, cell activity, cell proliferation, and alterations in gene expression associated with diminished regenerative outcomes. Importantly, these deficiencies in bone regeneration in skeletally mature Ts65Dn mice have implications to the adult T21 population as the last several decades have seen substantial increases in the average life span of T21 individuals. If the regenerative defects in Ts65Dn mice are recapitulated during bone healing in the T21 population, this could have profound consequences for this growing population.

## 1. Introduction

Down syndrome (DS) is the result of trisomy of human chromosome 21 (Hsa21) [1]. The prevalence of Trisomy 21 (T21) is rising with an occurrence of approximately 1:700 live births (12.8 [1]–15.55 [2] per 10,000 live births). The consequences of T21 are multifaceted and effect multiple organ systems across the lifespan, including the musculoskeletal system [3–11]. Skeletal consequences of T21 include short stature [12,13], early attainment of peak adult bone mass and subsequent early onset bone loss [10,14], and low bone mineral density (BMD) [3,6,14], all of which predispose the T21 population to fractures [15]. In addition, it is becoming clear that the T21 population also has a reduced capacity to repair bone, as a recent report demonstrated disrupted fracture healing in an infant with T21 [16]. Fractures are more prevalent in the aging T21 population, especially in females after the age of 50 [11]. Importantly, accelerated aging is a well-characterized and consistent hallmark of DS [17–20]. The atypical aging of individuals with T21 is characterized by a wide array of premature age-associated changes, including early onset menopause [21–23], thyroid dysfunction [24], vision [25] and hearing impairments [26], early onset Alzheimer’s disease (reviewed [17]), as well as premature degenerative cartilage and bone anomalies [3,15,27]. As such, developing a deeper understanding of the capacity for bone repair across the lifespan in T21 is a critical need.

There are multiple murine models of DS [1,5,28,29] that we and others have shown parallel the basal skeletal deficits observed in the T21 population, including the well-characterized Dp16(16)1Yey (Dp16) and Ts65Dn mouse models [4,30–34]. However, despite the many investigations of basal bone phenotypes in DS mice, there are few studies that investigate how the altered basal bone phenotypes translate to an injury environment [4,35]. Importantly, we have demonstrated impaired secondary fracture healing in 4-month-old Dp16 mice, characterized by reduced endochondral callus formation and complete fracture non-union [4]. In addition, using a standardized model of intramembranous bone regeneration, amputation of the distal digit tip, the terminal phalanx, P3, we reported sexually dimorphic P3 bone regeneration outcomes in young, 8-week-old Dp16 and Ts65Dn mice, with only male Ts65Dn mice exhibiting severe unresolved regeneration defects [35]. These P3 digit regeneration defects were associated with reduced osteoclast and osteoblast differentiation and resulted in attenuated P3 bone regeneration. Intriguingly, osteoclast and osteoblast differentiation were unaltered in Ts65Dn females, yet P3 bone regeneration was transiently enhanced [35]. Taken together, evidence suggests DS mice exhibit alterations in chondrocytes, osteoblasts, and osteoclasts during skeletal repair and regeneration.

**Fig. 1.**
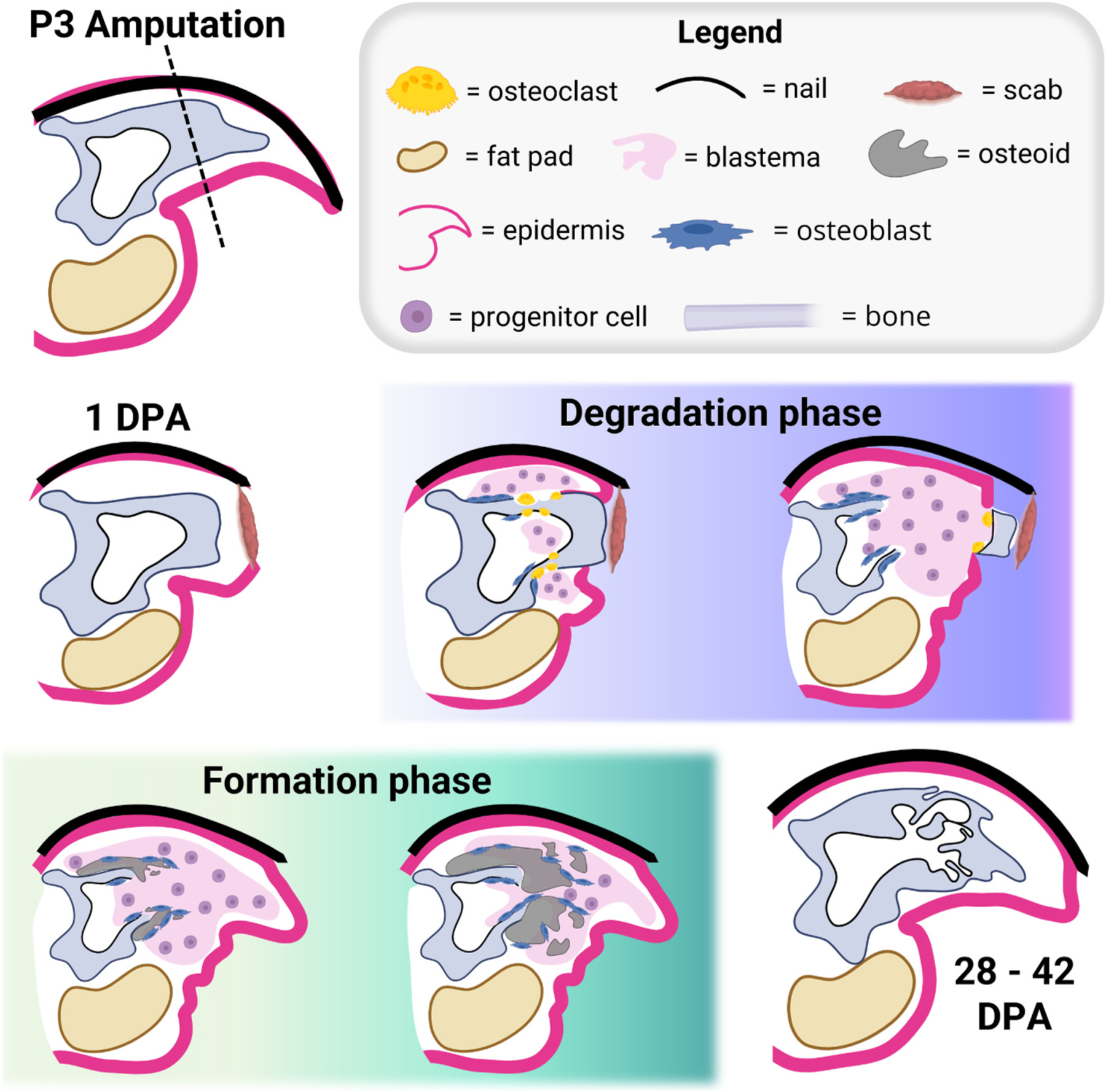
The P3 regeneration cascade. Distal P3 amputations are regenerative, and initiate a biphasic regenerative response first characterized by the degradation phase in which osteoclast resorption expels the necrotic bone fragment. Simultaneous with the degradation phase, the P3 blastema is initiated, and begins differentiating into bone *via* intramembranous ossification during the formation phase. At approximately 28–42 days post amputation (DPA), digit regeneration is complete. Distal is to the right, dorsal is to the top. Image created with BioRender.

While mammals have limited *de novo* regenerative ability following traumatic amputation, both young and adult mice [36–42] and humans [38,43–45] can regenerate their amputated distal digit tips. In mice, P3 amputation triggers an initial wound healing response characterized by osteoclast-mediated bone resorption that essentially “re-amputates” the P3 stump, thus facilitating the expulsion of the necrotic bone fragment [36,46]. During P3 degradation, formation of a structure called the blastema occurs. The blastema is a transient population of proliferating cells that differentiate and give rise to all the amputated structures, including bone [36,38,47]. Blastemal osteoprogenitors directly differentiate into bone, thus P3 regeneration is an endogenous model of intramembranous ossification. Following P3 degradation and subsequent wound closure, blastemal osteoblasts produce osteoid originating at the stump/blastema interface and extend toward the distal tip [42]. Bone mineralization and continued bone regeneration facilitate restoration of the amputated skeletal length by 28–42 days post amputation (DPA) [36,48]. P3 regeneration is robust but imperfect, as enhanced ossification on the dorsal-ventral axis [48] results in a characteristic overshoot in bone volume compared to the unamputated P3 bone. Nevertheless, the biphasic P3 regeneration cascade is a powerful model that allows for the temporal investigation of osteoclast and osteoblast recruitment and activity *in vivo* (Fig. 1), without an intermediate cartilaginous callus step. Thus, P3 regeneration is distinct from endochondral bone repair (*i.e.* unstabilized fracture healing) due to the lack of the cartilaginous step [48]. Considering that fracture healing is inhibited at the chondrogenic phase in Dp16 DS mice [4], and that both osteoclasts and osteoblasts can be investigated during P3 regeneration, the current studies took advantage of the P3 intramembranous ossification model to investigate bone degradation and osteogenesis in DS.

Accelerated aging is a hallmark of DS [17–19], therefore we questioned to what extent attaining skeletal maturity impacts P3 bone regeneration in DS mice. Ts65Dn DS mice are trisomic for approximately 75 % of *Hsa21*-homologous genes [28,49], and have been shown to exhibit skeletal deficits across the lifespan [4,30,31,33,34,50–52]. Due to these known skeletal alterations in Ts65Dn mice, P3 regeneration was investigated in 6-month-old Ts65Dn mice, which is 3 months beyond peak adult bone mass [30]. Here, we demonstrate that the P3 intramembranous bone regeneration anomalies observed in young (2-months-old) Ts65Dn male mice are exacerbated by 6-months-old, and that bone resorption deficits are now observed in skeletally mature female Ts65Dn mice that were absent at 2 months of age. Collectively, these data demonstrate that adult skeletally mature Ts65Dn mice show fundamental *in vivo* deficits in progenitor cell differentiation, cell activity, cell proliferation, as well as alterations in gene expression associated with poor regenerative outcomes.

## 2. Methods

### 2.1. Animals and surgery

Trisomic B6EiC3Sn *a*/*A*-Ts(17 [16])65Dn/J (Ts65Dn; strain #001924) female mice and Wildtype (WT) euploid male B6EiC3SnF1/J (strain #001875) mice were purchased from The Jackson Laboratory (Bar Harbor, ME) and bred at the Texas A&M Institute for Genomic Medicine (TIGM). At TIGM, all mice had *ad libitum* access to standard rodent chow and water. All P3 baseline and P3 amputation studies were carried out on 6-month-old animals, using Ts65Dn mice and WT littermates as controls. P3 amputation has been described in detail [36,53]. For P3 amputation, mice were anesthetized using isoflurane, with an initial dose of 3 % and maintained at 2 % over the duration of the surgery. On each mouse, digits 2 and 4 of both hind paws were amputated at the standard distal P3 amputation plane [53,54], using up to 4 digits per mouse. All animals were treated similarly, regardless of genotype. All animal use and techniques were in compliance with the standard operating procedures of the Texas A&M University IACUC.

### 2.2 Digit processing, histological staining, immunohistochemistry, and image analysis

Digits were harvested at 7, 10, and 14 days post amputation (DPA), as well as unamputated digits. Following digit harvest, digits were fixed in 10 % Neutral Buffered Formalin for 24–96 h at room temperature with gentle shaking. After fixation, digits were decalcified using Decalcifier 1 (Surgipath, Leica Biosystems, Richmond, IL) for 20–24 h at room temperature with gentle shaking. Digits were processed through a graded ethanol series, xylenes, and paraffin prior to paraffin embedding. All digits were sectioned at 4 μm thickness. For all histological staining and immunohistochemistry, slides were incubated at 65 °C for 45 min followed by incubation at 37 °C for 15 min. For all immunostaining, antigen heat retrieval was carried out by incubating slides in 1× Tris-EDTA (pH 9.0) for 6 h at 65 °C to maintain tissue morphology. After antigen retrieval, slides were incubated using Protein Block Solution (DAKO) for 1 h at room temperature. After blocking, the slides were incubated in primary antibody overnight at 4 °C. The following primary antibodies were used in this study: rabbit anti-Cathepsin K (CTSK) (Abcam; ab187647; 1:300 dilution), monoclonal mouse anti-Proliferating Cell Nuclear Antigen (PCNA) (Abcam; ab29; 1:1000 dilution), and monoclonal rabbit anti-Runx2 (RUNX2) (Abcam; ab192256; 1:400 dilution). Slides were then incubated in the following secondary antibodies for 45 min at room temperature: Alexa Fluor goat anti-mouse 647 IgG secondary antibody (Invitrogen, A21235, 1:500 dilution) and the Alexa Fluor goat anti-rabbit 488 IgG secondary antibody (Invitrogen, A11008, 1:500 dilution). Samples were then counterstained with DAPI for 5 min at room temperature. To quantify CTSK^+^, Runx2^+^, and PCNA^+^ cells, slides were imaged using the Olympus VS120 microscope with a Hamamatsu ORCA-Flash 4.0 camera using *VS*-ASW Fl2.8 software (Olympus), with image processing using Fiji [55] and the BIOP VSI reader [56] as described [42]. For general histological imaging and generation of images for erosion perimeter and total bone perimeter quantification, slides were imaged using the Olympus BX60 microscope and Olympus Dp72 camera, with image processing using the Dp2-BSW software (Olympus America Inc., Center Valley, PA). For fluorescent imaging, slides were imaged using the Olympus BX61 fluorescence deconvolution microscope using the Slidebook software (Intelligent Imaging Innovation Inc., Denver, CO). All tissue assessments were carried out blind to sex and genotype. Quantification of osteoclasts and osteoblasts has been described [35,46]. Briefly, osteoclasts were identified as CTSK^+^ cells containing three or more nuclei and localized to the bone surface, and quantified over total P3 bone perimeter. Mallory trichrome stained serial sections were utilized to quantify erosion perimeter and total bone perimeter. Runx2^+^, PCNA^+^, and DAPI stained nuclei were manually counted, and Runx2 and PCNA were normalized to total DAPI nuclei.

### 2.3. Microcomputed tomography (μCT) scanning

*In vivo* μCT scanning was carried out on P3 digits prior to amputation and at various time points throughout regeneration as previously described [35,42,46,48,53,54,57] using the vivaCT 40 (SCANCO Medical, Wayne Pennsylvania). P3 was scanned at a voxel size of 10.5 μm, at 55kVp, 145 μA, 300 msec integration time, with 1000 projections per 180° using continuous rotation, following standard ASBMR practices [58]. The scanning ROI was approximately 2 mm, and μCT dose index (CTDI) was 960 mGy per scan [46]. Creation of images and analysis of bone volume and length was carried out using the BoneJ Plugin for Image J (Fiji) [59] as described [53]. Pseudo-coloring of regenerated digits was performed using PowerPoint.

### 2.4. qRT-PCR Analysis

Total RNA was extracted from Ts65Dn male (12 pooled digits), WT male (12 pooled digits), Ts65Dn female (12 pooled digits), and WT female (12 pooled digits) at 7 and 10 DPA using the RNeasy Plus Micro Kit (Qiagen), following the manufacturer’s instructions. RNA quantification and quality was performed using nanodrop ratios of 260/280 and 260/310. After extraction, qRT-PCR was performed in triplicate with the SuperScript III Platinum One-Step qRT-PCR Kit w/Rox using the Eppendorf Realplex machine. The following Applied Biosystem Taqman primer sets (Thermo Fisher) were used: RankL (Tnfsf11) (Mm00441906_m1); Rank (Tnfrsf11a) (Mm00437132_m1); OPG (Tnfrsf11b) (Mm00435454_m1); Prx1 (Prrx1) (Mm00440932_m1); Runx2 (Mm00501584_m1); Sp7 (Osterix) (Mm04209856_m1); Bmp2 (Mm01340178_m1); Bmp4 (Mm00432087_m1); Bmp7 (Mm00432102_m1); Dlx5 (Mm07296590_m1), and Noggin (Mm01297833_s1). Gene expression levels were normalized to the levels of the housekeeping gene, ribosomal protein L12 (RPL12). Gene expression levels were analyzed using GraphPad PRISM (GraphPad Software, La Jolla, CA; version 9.5.1 (2023)), using an unpaired *t*-test.

### 2.5. Statistics

Statistical tests were performed using GraphPad PRISM, version 9.5.1 (2023), (GraphPad Software, La Jolla, CA). For the repeated measures of bone volume and length during P3 regeneration, a Two-way ANOVA and Sidak’s multiple comparisons test was performed. Baseline P3 volume and length, as well as qPCR analyses were analyzed using an unpaired *t*-test. Immunostaining was analyzed using a Two-way ANOVA with Tukey’s multiple comparisons test. Wound closure was assessed by assessing open *vs* closed wounds at every time point using the Fisher’s exact test. *P* values for wound closure analyses indicate no significance (^ns^P > 0.05) across all assessed time points.

## 3. Results

### 3.1. Sexually dimorphic bone degradation and bone regeneration responses in 6-month-old Ts65Dn mice

**Fig. 2.**
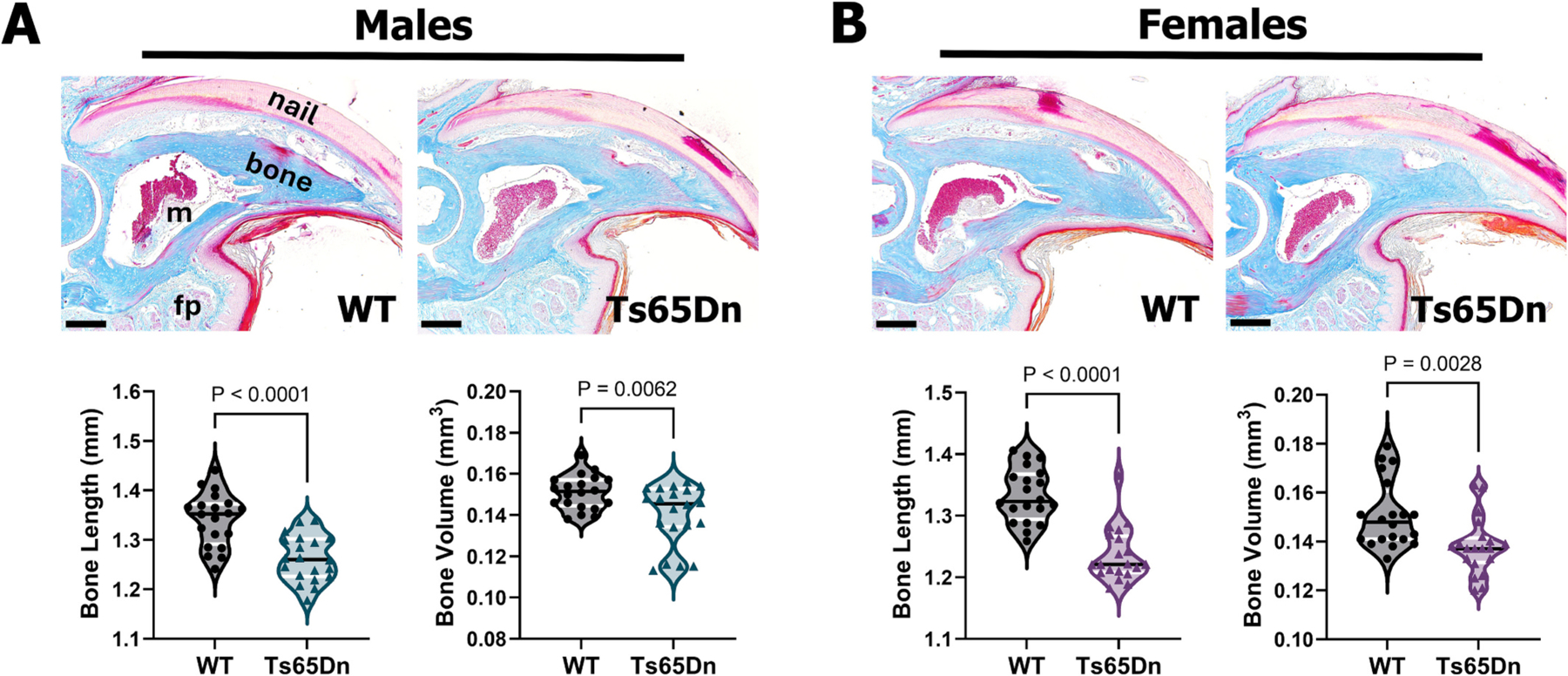
Baseline unamputated P3 bone length and volume are attenuated in male and female 6-month-old Ts65Dn mice. (A) Mallory trichrome staining of representative WT and Ts65Dn male unamputated P3 digits. Bone length and volume are significantly reduced in Ts65Dn males compared to the WT controls. (B) Mallory trichrome staining of representative WT and Ts65Dn female unamputated P3 digits. Bone length and volume are significantly reduced in Ts65Dn females as compared to the WT controls. Distal is to the right, dorsal is to the top. Violin plots are showing individual data points, medians and 1st and 3rd quartiles, Student’s *t*-test. M = marrow. Fp = Fat pad. Scale bars = 200 μm.

We and others have previously demonstrated skeletal deficits in Ts65Dn mice across the lifespan [4,30–34,50–52]. Skeletal alterations were also observed in the unamputated terminal phalanx (P3) bone of young (8-week-old) male and female Ts65Dn mice, with P3 exhibiting reduced bone volume and length at baseline [35]. Moreover, we showed that P3 regeneration was significantly reduced in young Ts65Dn males but was transiently enhanced in young Ts65Dn female mice [35]. Given that DS is an example of atypical aging [17–19], we questioned to what extent attaining skeletal maturation (6-month-old mice, equivalent to a 30-year-old human [60]) impacts P3 bone regeneration in Ts65Dn mice. To this end, histological analysis and μCT scanning was performed on unamputated P3 digits of 6-month-old male and female Ts65Dn mice and euploid WT controls to determine the baseline phenotype of the P3 digit (Fig. 2A). Histological analysis did not reveal apparent alterations of the P3 bone, the central marrow compartment, the ventral fat pad, or the surrounding nail organ between Ts65Dn mice and WT controls (Fig. 2). Conversely, as in 8-week-old mice [35] μCT analysis identified significantly reduced P3 bone length and volume in Ts65Dn males (*n* = 18 digits) and Ts65Dn females (*n* = 20 digits) compared to WT males (n = 20 digits) and WT females (n = 20 digits), respectively (Fig. 2). We next questioned if Ts65Dn mice and WT controls exhibited differences in baseline body weight at 6-months-old. Both males (WT (*n* = 11 mice) and Ts65Dn (*n* = 12 mice)) and females (WT (n = 12 mice) and Ts65Dn (*n* = 15 mice)) showed no differences in body weight (Supplementary Fig. 1).

**Fig. 3.**
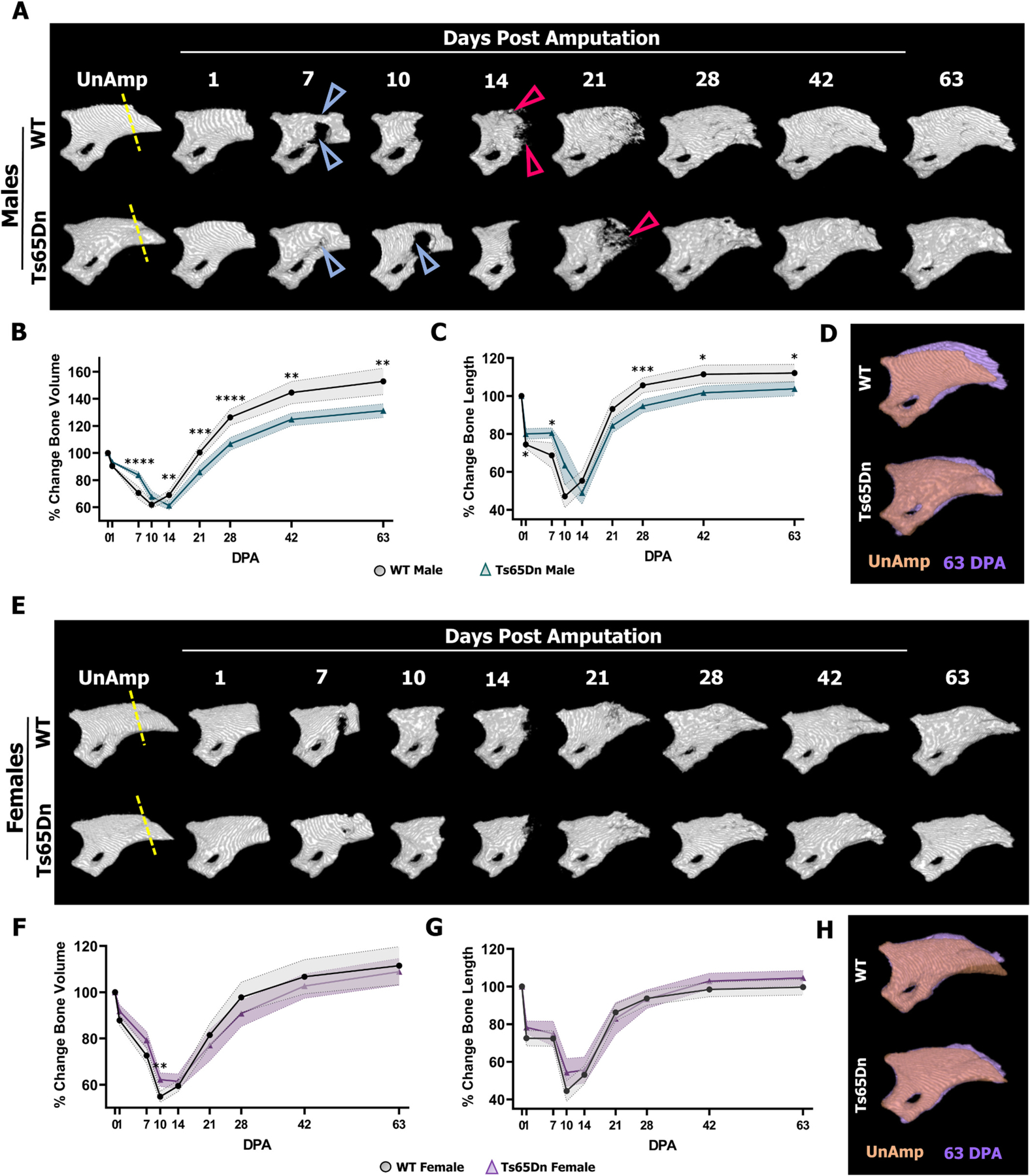
6-month-old Ts65Dn mice exhibit diminished bone degradation and only males demonstrate attenuated bone regeneration. (A) Representative μCT renderings of a single male WT (top) digit and a single male Ts65Dn DS (bottom) digit during the regeneration cascade. The yellow line represents the amputation plane on the unamputated digit, the light blue arrowheads represent the degradation response with osteoclast resorption pits, and the red arrowheads represent the regenerated bone islands during regeneration. The P3 degradation response expels the necrotic bone and is following by regeneration of the amputated structures *via* intramembranous ossification. (B) Ts65Dn DS males show delayed degradation at 7 (****P < 0.0001) and 14 (** *P* = 0.0065) days post amputation (DPA) and attenuated regeneration at 21 (***P = 0.0006), 28 (****P < 0.0001), and 42 (**P = 0.0013) that does not resolve by 63 DPA (***P = 0.0028). (C) Ts65Dn males have reduced bone length degradation at 7 (*P = 0.0188) DPA and reduced gains in length at 28 (***P = 0.0008), 42 (*P = 0.0156) and 63 DPA (*P = 0.0471) compared to the WT controls. (D) Pseudo-colored overlay of the unamputated and 63 DPA μCT rendering. WT males exhibit the characteristic bone volume overshoot at 63 DPA while the Ts65Dn males show an attenuated response. (E) Representative μCT renderings of a single female WT (top) P3 bone and a single female Ts65Dn (bottom) P3 bone over the duration of regeneration. A similar degradation and regeneration response occurs to restore bone length and volume after amputation. (F) Female Ts65Dn DS mice have decreased degradation at 10 DPA (**P = 0.0024) but comparable regeneration to WT females. (G) Ts65Dn females have similar bone length measurements to WT females over the duration of P3 regeneration. (H) Pseudo-colored overlay of the unamputated and 63 DPA μCT rendering. Both DS and WT females lack the characteristic bone volume expansion from regeneration. Distal is to the right, dorsal is to the top. (For interpretation of the references to colour in this figure legend, the reader is referred to the web version of this article.)

To investigate P3 bone regeneration in 6-month-old DS mice, P3 amputation [36,53] (dashed lines in Fig. 3A, E) was carried out on male and female Ts65Dn mice and WT controls, followed by longitudinal *in vivo* μCT scanning (Fig. 3A, E). P3 amputation removes approximately 10 % of the bone volume and 30 % of the bone length, and triggers a biphasic regeneration response characterized by osteoclast-mediated bone degradation to remove the necrotic stump tip, followed by intramembranous ossification to restore the entire P3 element [36,38,42,46,48,53,54,61]. In WT males (n = 20 digits), P3 bone degradation was evident by 7 days post amputation (DPA), characterized by bone resorption proximal to the amputation plane (Fig. 3A, blue arrowheads) and an associated decrease in bone volume and length (Fig. 3A–C). By 10 DPA, resorption culminated with stump re-amputation, resulting in the remaining P3 bone comprising approximately 60 % volume and 50 % length of the original unamputated bone (Fig. 3A–C). Conversely, Ts65Dn males (*n* = 18 digits) showed attenuated degradation evident by minimal pitting at 7 DPA and significantly less degradation at 7 DPA compared to WT males, and the culmination of resorption at 14 DPA (Fig. 3A–C). Given that we had previously demonstrated that P3 bone degradation is only transiently reduced in young Ts65Dn male mice [35], these findings suggest that P3 resorption defects are exacerbated in skeletally mature male Ts65Dn mice. The onset of bone mineralization (Fig. 3A, red arrowheads) was apparent by 14 DPA in WT males and 21 DPA in Ts65Dn males (Fig. 3A–C). Ts65Dn males progress through the regeneration phase yet showed significantly reduced gains in bone volume and length at all time points assayed compared to WT males (Fig. 3A–C), providing evidence that the characteristic overshoot in P3 volume was attenuated in Ts65Dn males. The overshoot in P3 bone volume and the restoration of amputated skeletal length is associated with periosteal bone formation [48], and indeed, WT male digits exhibited an expansion of bone localized to the periosteal compartment by 63 DPA that was comparatively minimal in Ts65Dn male digits (Fig. 3D). Our previous P3 bone regeneration studies in young Ts65Dn females demonstrated an equivalent bone degradation phase between Ts65Dn and WT females, and temporarily enhanced P3 bone regeneration at 14 and 21DPA [35]. In 6-month-old female mice, Ts65Dn digits (*n* = 20 digits) exhibited reduced bone degradation associated with increased bone volume at 10 DPA yet progressed through bone regeneration comparable to WT females (n = 20 digits) (Fig. 3E–G), thus the transiently enhanced bone regeneration observed in young female Ts65Dn mice was absent by 6 months of age. Intriguingly, unlike males, both Ts65Dn and WT females did not exhibit a robust overshoot in bone volume by 63 DPA and likewise did not demonstrate overt expansion of the periosteal compartment by 63 DPA (Fig. 3F, H, compared to 3B and D). Lastly, because P3 regeneration is dynamic and involves the degradation of bone and the subsequent rebuilding of bone, we questioned if the fully regenerated bone at 63DPA in Ts65Dn mice was comparable to WT controls at 63 DPA. At 63 DPA, Ts65Dn males (n = 12 digits) exhibited reduced bone length and volume compared to WT males (n = 20 digits) (Supplementary Fig. 2A), whereas female Ts65Dn mice (n = 20 digits) showed comparable bone length and decreased bone volume compared to WT females (n = 20 digits) (Supplementary Fig. 2B). Collectively, these findings demonstrate that by 6 months of age, skeletally mature Ts65Dn mice have sexually dimorphic deficits on both bone degradation and bone regeneration.

### 3.2. Attenuated osteoclast and osteoblast differentiation is associated with dysregulated gene expression

**Fig. 4.**
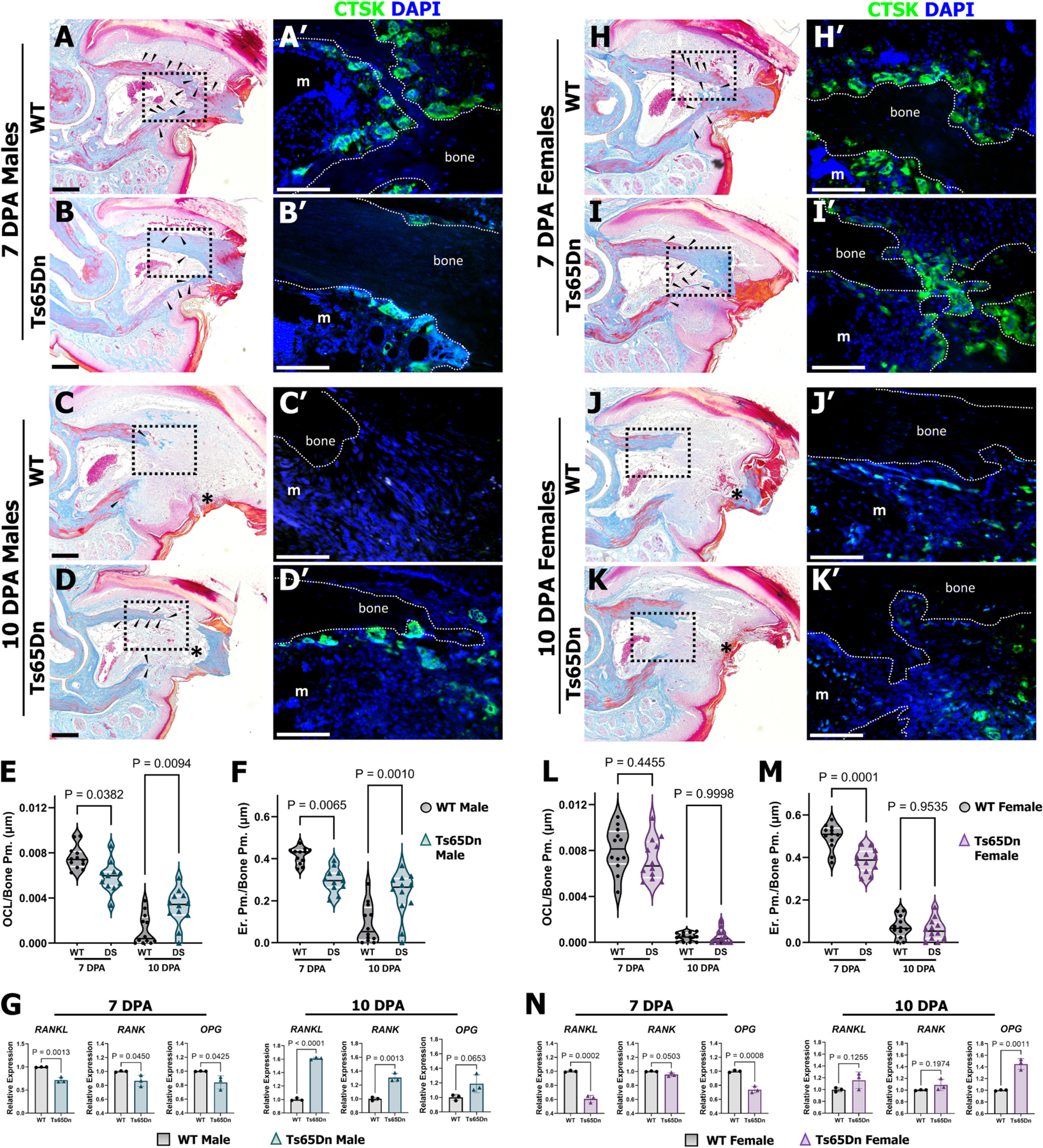
6-month-old Ts65Dn mice have sexually dimorphic degradation outcomes at 7 and 10 DPA. (A-A’) Representative sections of a WT male P3 bone at 7 DPA. (A) Mallory trichrome staining at 7 DPA shows osteoclast resorption pits (arrowheads). (A’) Immunostaining for Cathepsin K (CTSK) illustrates osteoclasts lining the bone. (B–B’) Representative sections of a Ts65Dn male P3 bone at 7 DPA. (B) Mallory trichrome staining shows osteoclast resorption pits (arrowheads) at 7 DPA. (B′) CTSK immunostaining illustrates decreased osteoclast number at 7 DPA. (C–C’) Representative sections of WT males at 10 DPA. (C) Mallory trichrome staining at 10 DPA showing minimal osteoclast resorption pits (arrowheads). (C’) Immunostaining for CTSK shows osteoclasts are nearly absent at 10 DPA in WT males. (D–D’) Representative serial sections of Ts65Dn males at 10 DPA. (D) Mallory trichrome staining at 10 DPA demonstrating osteoclast resorption pits (arrowheads), and the remaining severed necrotic bone not yet expelled in Ts65Dn males. (D′) CTSK immunostaining illustrates osteoclasts still present at the P3 stump at 10 DPA. (E) CTSK^+^ osteoclasts quantified at 7 and 10 DPA show attenuated osteoclasts in Ts65Dn males at 7 DPA and delayed removal of osteoclasts at 10 DPA. (F) Quantification of erosion perimeter illustrates diminished eroded bone surface at 7 DPA in Ts65Dn males. (G) Expression of osteoclast markers (measured by qPCR and normalized to *Rpl12) Rankl, Rank,* and *Opg* were attenuated in Ts65Dn males at 7 DPA but *Rankl* and *Rank* were upregulated at 10 DPA. (H–H’) Representative serial sections of a WT female P3 bone at 7 DPA. (H) Mallory trichrome staining at 7 DPA shows osteoclast resorption pits (arrowheads). (H’) Immunostaining for CTSK illustrates osteoclasts lining the bone. (I–I’) Representative serial sections of a Ts65Dn female P3 bone at 7 DPA. (I) Mallory trichrome staining shows osteoclast erosion pits (arrowheads) at 7 DPA. (I′) CTSK immunostaining illustrates similar osteoclast number on the degrading bone at 7 DPA. (J–J’) Representative serial sections of a WT female at 10 DPA. (J) Mallory trichrome staining at 10 DPA showing fewer osteoclast erosion pits present on the bone perimeter. (J’) Immunostaining for CTSK shows osteoclasts are absent on the bone perimeter at 10 DPA in WT females. (K-K’) Representative serial sections of a Ts65Dn female at 10 DPA. (K) Mallory trichrome staining at 10 DPA showing fewer osteoclast erosion pits on the bone perimeter. (K’) CTSK immunostaining illustrates osteoclasts absent at the P3 bone perimeter at 10 DPA. (L) CTSK+ osteoclasts quantified at 7 and 10 DPA show comparable osteoclasts in WT and Ts65Dn females at 7 and 10 DPA. (M) Quantification of erosion perimeter illustrates attenuated eroded bone surface at 7 DPA in Ts65Dn females. (N) Expression of osteoclast markers *Rankl* and *Opg* were attenuated in Ts65Dn females at 7 DPA whereas *Opg* was upregulated at 10 DPA. Gene expression shown as bar graphs with control set to 1. Distal is to the right, dorsal is to the top. Arrowheads represent osteoclast resorption pits along the bone perimeter. Violin plots (E, F, L, M) showing individual data points, medians and 1st and 3rd quartiles, two-way ANOVA. Student’s *t*-test for bar graphs, and error bars indicate SD. M; marrow; OCL, Osteoclast; Bone Pm., Bone Perimeter; Er. Pm, Erosion Perimeter. Immunohistochemical stained samples counterstained with DAPI (blue). Scale bars: immunohistochemistry = 50 μm; histology = 200 μm. (For interpretation of the references to colour in this figure legend, the reader is referred to the web version of this article.)

The utility of the biphasic P3 regeneration model is that it enables the temporal investigation of osteoclast-driven bone degradation and subsequent osteoblast-driven intramembranous ossification *in vivo* (Fig. 1). Therefore, to better understand the attenuated degradation response observed in 6-month-old Ts65Dn mice (Fig. 3), we harvested amputated digits during the degradation phase (7 DPA) and the approximate resolution of the degradation phase (10 DPA) from male and female Ts65Dn mice and WT controls. Histological staining of WT male digits (*n* = 12 digits) showed osteoclast resorption pits along the dorsal and ventral periosteal surface and associated with the distal endosteal surface (Fig. 4A, arrowheads), whereas Ts65Dn male digits exhibited a comparatively smoother bone surface with minimal bone resorption (Fig. 4B, arrowheads). In line with this, immunostaining for the osteoclast marker, Cathepsin K (CTSK), revealed significantly reduced osteoclasts and an associated reduced erosion perimeter in Ts65Dn males compared to WT males at 7 DPA (Fig. 4A’, B’, E, F). By 10 DPA, WT male digits (*n* = 12) had largely resolved the degradation phase, with minimal pitting on the bone surface (Fig. 4C, arrowheads), few osteoclasts (Fig. 4C’, E), and diminished eroded bone surface (Fig. 4F). In contrast, eroded bone surface (Fig. 4D, arrowheads) and osteoclasts were still present in Ts65Dn males (*n* = 11 digits, Fig. 4D–E). To tease apart the altered osteoclast response in Ts65Dn males, we assayed by real time quantitative PCR (qPCR) the gene expression of *Rankl*, involved in osteoclast formation and activation, *Rank*, involved in the stimulation of osteoclast differentiation, activation, and survival, and *Opg*, involved in the inhibition of osteoclast formation and activation and thus the promotion of bone formation (reviewed in [62]) (Fig. 4G). At 7 DPA, *Rankl*, *Rank*, and *Opg* were significantly reduced in Ts65Dn males compared to WT males (Fig. 4G), consistent with the observed fewer osteoclasts and erosion perimeter at 7DPA. By 10 DPA, Ts65Dn males showed enhanced *Rankl* and *Rank*, and no difference in *Opg* expression compared to WT males (Fig. 4G), and thus consistent with the persistence of osteoclasts and eroded bone surface (Fig. 4D). In female mice, while no difference was observed in osteoclast recruitment, Ts65Dn females (*n* = 12 digits) showed reduced erosion perimeter compared to WT females (n = 12 digits) at 7 DPA (Fig. 4H–I’, L, M), suggestive of diminished osteoclast activity. By 10 DPA, both osteoclasts and erosion perimeter were markedly reduced, with no significant changes observed between WT (n = 12 digits) and Ts65Dn females (n = 12 digits) (Fig. 4J–M). Congruent with the diminished erosion perimeter at 7 DPA (Fig. 4M), expression levels for *Rankl* and *Opg* mRNAs were reduced in Ts65Dn females at 7 DPA (Fig. 4N). By 10 DPA, no differences were observed in *Rankl* and *Rank* expression, whereas *Opg* was upregulated in Ts65Dn females (Fig. 4N). Given that osteoclast recruitment and activity is attenuated in young Ts65Dn males yet is unaltered in young Ts65Dn females [35], these findings demonstrate the persistence of *in vivo* osteoclast defects in DS males and the gain of osteoclast defects in DS females by 6 months of age.

**Fig. 5.**
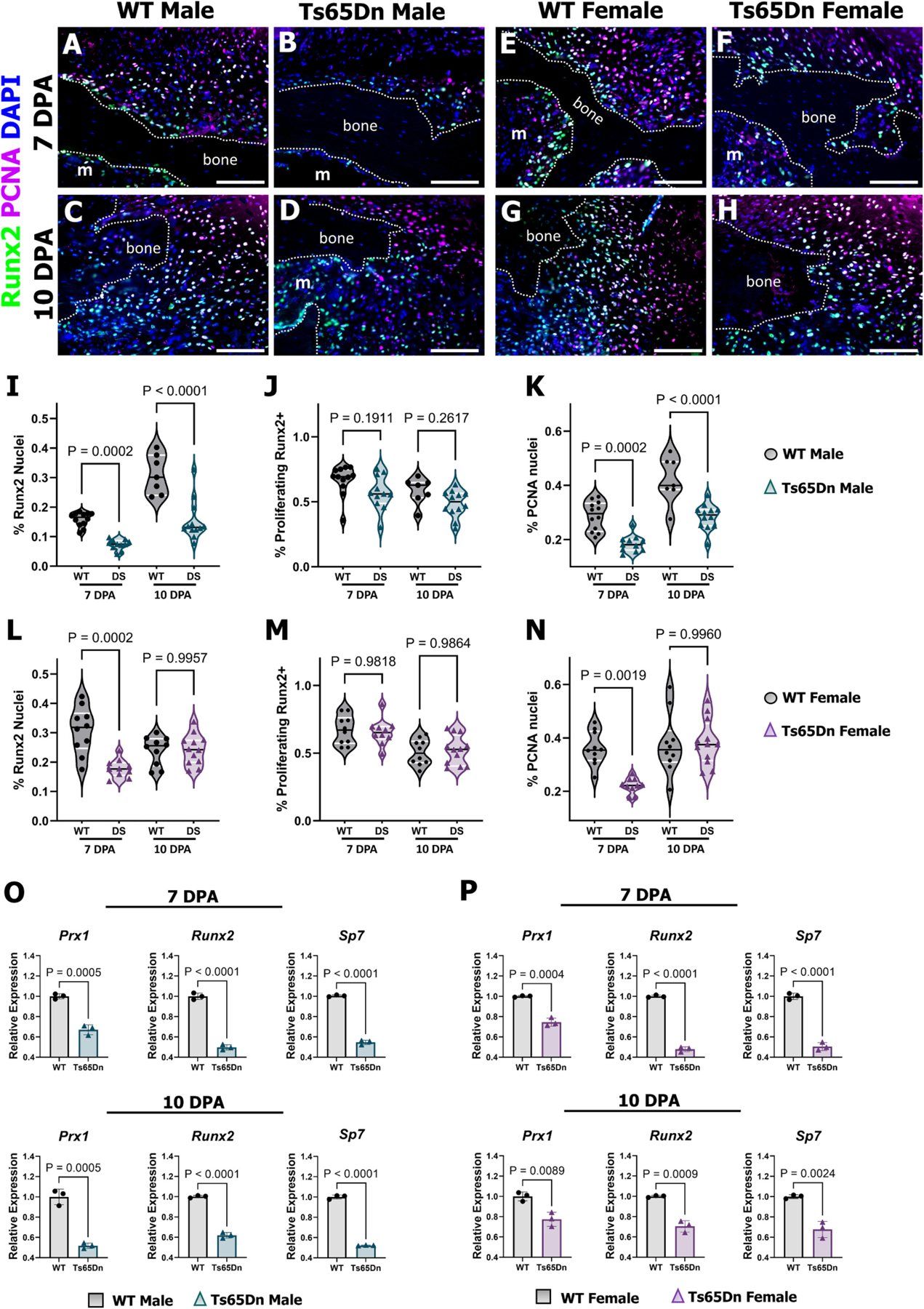
Shared fundamental defect in the pool of progenitors in 6-month-old Ts65Dn DS mice manifests as reduced bone regeneration in males but not in females. (A) Co-immunostaining for the osteoprogenitor marker Runx2 and Proliferating Cell Nuclear Antigen (PCNA) at 7 DPA in WT males shows an abundance of Runx2^+^ and PCNA^+^ cells localized to the bone stump compared to (B) Ts65Dn males at 7 DPA that show similar localization but reduced number of Runx2^+^ and PCNA^+^ cells. (C) Co-immunostaining at 10 DPA for WT males illustrates an abundance of Runx2^+^ and PCNA^+^ cells compared to (D) Ts65Dn males. (E) WT female representative section at 7 DPA illustrates an abundance of Runx2^+^ and PCNA^+^ cells at the bone and endosteal/marrow perimeter compared to (F) Ts65Dn females. (G) At 10 DPA, co-immunostaining reveals equivalent Runx2^+^ and PCNA^+^ cells in WT females and (H) Ts65Dn females at 10 DPA. (I–K) Ts65Dn males have reduced Runx2^+^ cells at 7 and 10 DPA (I), comparable co-immunostaining of Runx2^+^/PCNA^+^ cells at 7 and 10 DPA (J), and reduced PCNA^+^ nuclei at 7 and 10 DPA (K). (L-N) Female Ts65Dn mice have attenuated Runx2^+^ nuclei at 7 DPA (L), similar Runx2^+^/PCNA^+^ to WT females at 7 and 10 DPA (M), and reduced PCNA^+^ nuclei at 7 DPA (N). (O–P) *Prx1, Runx2,* and *Sp7* expression (measured by qPCR and normalized to *Rpl12*, shown as bar graphs with control set to 1) was reduced at 7 and 10 DPA in Ts65Dn males (O) and females (P) compared to their WT counterparts. Violin plots (I–N) showing individual data points, medians and 1st and 3rd quartiles, two-way ANOVA. Bar graphs calculated with Student’s t-test, and error bars indicate SD. Distal is to the right, dorsal is to the top. M, marrow. Samples counterstained with DAPI. Scale bars = 50 μm.

To interrogate osteogenic differentiation and proliferation in 6-month-old Ts65Dn mice, co-immunostaining for the osteoprogenitor marker Runx2 and Proliferating Cell Nuclear Antigen (PCNA) was performed on digits at 7 and 10 DPA. At 7 DPA in WT males (n = 12 digits), Runx2^+^ cells were localized to the endosteal/marrow region and associated with the expansion of the periosteal compartment (Fig. 5A), whereas Ts65Dn males showed a comparable osteoprogenitor localization but with significantly fewer Runx2^+^ cells (Fig. 5B, I). By 10 DPA, Runx2^+^ cells were again abundant in WT males (*n* = 7) compared to Ts65Dn males (n = 12) (Fig. 5C, D, I). Importantly, osteoprogenitor proliferation was not altered in DS, as the percentage of Runx2^+^/PCNA^+^ cells were comparable between males at 7 DPA and 10 DPA despite reduced overall cell proliferation at the same time points (Fig. 5J, K). Intriguingly, while no differences in bone regeneration were observed by μCT analysis between Ts65Dn and WT females, 7 DPA Ts65Dn female digits exhibited reduced Runx2^+^ cells compared to WT controls yet showed no differences in the percentage of osteoprogenitors by 10 DPA (Fig. 5E–H, L). Osteoprogenitor proliferation was not altered at 7 and 10 DPA, whereas overall proliferation was reduced at 7 DPA in Ts65Dn females, yet this was resolved by 10 DPA (Fig. 5M, N). To gain a deeper understanding of intramembranous bone cell differentiation in DS, we assayed gene expression at 7 and 10 DPA by qPCR. Osteoblast differentiation is characterized by *Prx1*^+^ mesenchymal progenitors differentiating into *Runx2*^+^ osteoprogenitors, that then further mature into *Sp7* (Osterix) expressing osteoblasts [63]. At 7 and 10 DPA, expression levels of *Prx1*, *Runx2*, and *Sp7* were diminished in both Ts65Dn males and females compared to their WT counterparts (Fig. 5O, P). While these findings correlate to the Runx2 immunostaining in males at 7 and 10 DPA and in females at 7 DPA, they are puzzling for Ts65Dn females given their similar Runx2^+^ and PCNA^+^ cells at 10DPA and lack of μCT bone regeneration defects from 10 to 63DPA (Fig. 3). These data suggest a shared initial defect in the pool of progenitors in 6-month-old Ts65Dn mice that subsequently manifests as reduced osteoblast number and bone regeneration in males but not in females.

**Fig. 6.**
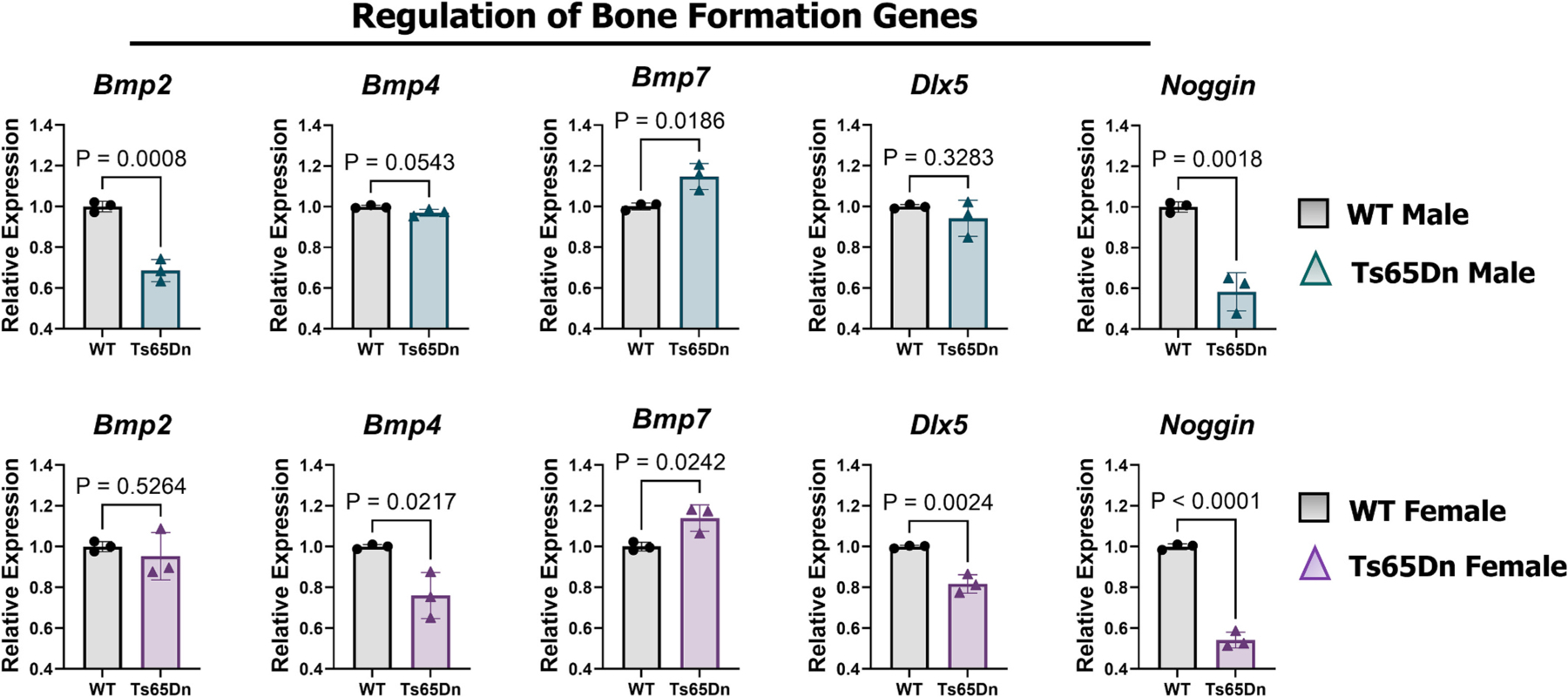
Ts65Dn males and females show dysregulated bone formation genes at 10 DPA. Gene expression analysis using qRT-PCR of bone formation genes in male and female WT and Ts65Dn mice at 10 DPA, shown as bar graphs with control set to 1. Gene expression was normalized to Rpl12. Student’s t-test, and error bars indicate SD.

The P3 blastema is a transient structure that facilitates P3 regeneration, and is characterized by enhanced *Dlx5* [64], *Bmp2*, *4*, and *7* expression [65]. Therefore, we assayed these bone formation genes at 10 DPA in Ts65Dn males and females and WT controls (Fig. 6). Ts65Dn males showed reduced *Bmp2* expression, enhanced *Bmp7* expression, and equivalent *Bmp4* and *Dlx5* expression compared to WT males (Fig. 6). Expression of the BMP2, 4, and 7 inhibitor, *Noggin*, was significantly downregulated in Ts65Dn males (Fig. 6). Ts65Dn females showed equivalent *Bmp2*, reduced *Bmp4* and *Dlx5*, and elevated *Bmp7* expression compared to WT females (Fig. 6). Like their male counterparts, Ts65Dn females exhibited reduced expression of *Noggin* (Fig. 6). Taken together, these findings demonstrate a complex dysregulation of bone formation genes in DS, associated with the promotion of bone regeneration in females and the attenuation of bone regeneration in males (Fig. 3).

### 3.3. Wound closure rate is not impacted in 6-month-old Ts65Dn mice

**Fig. 7.**
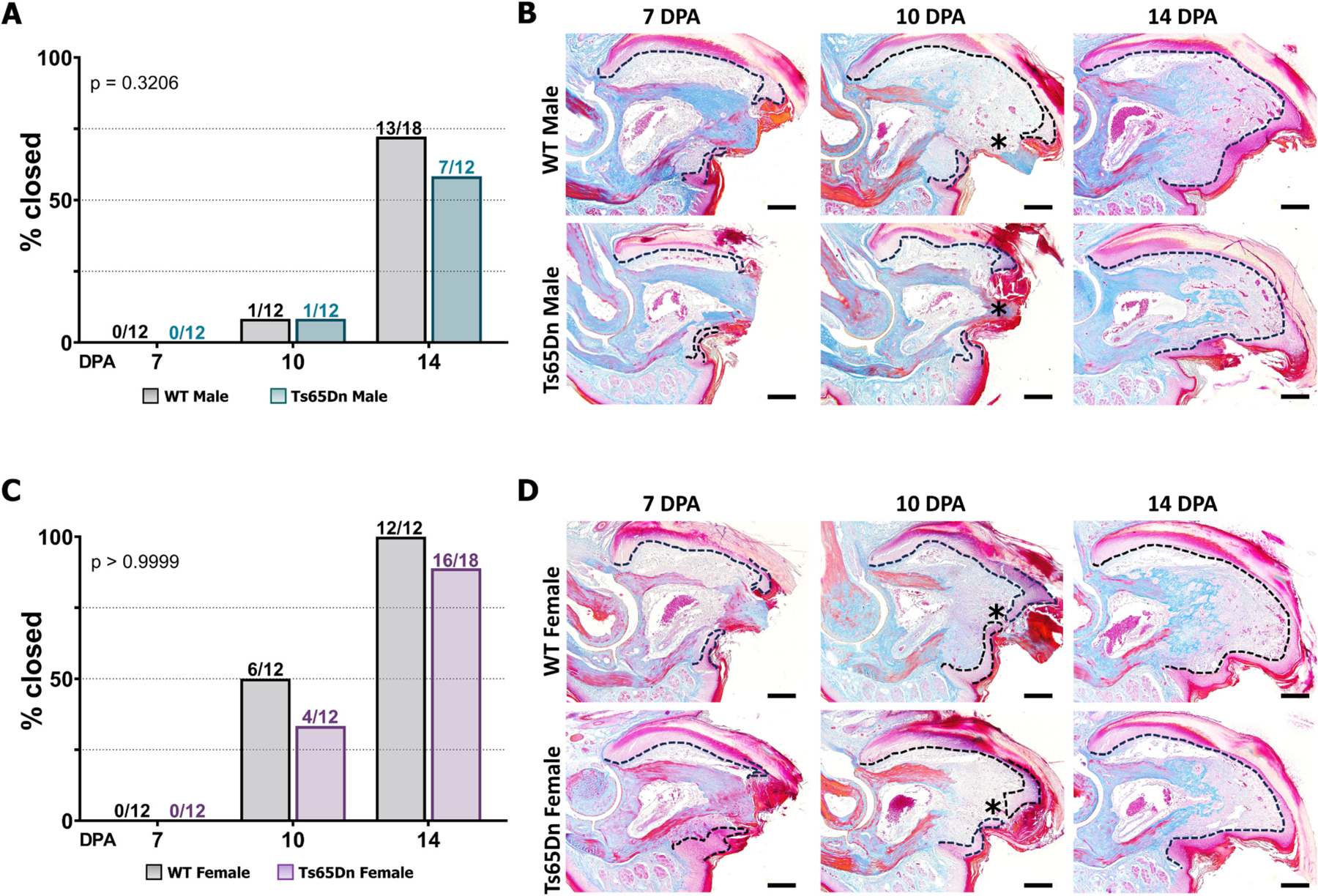
Wound closure is unaltered in 6-month-old Ts65Dn mice. (A) Number and percentage of digits with closed wounds at 7, 10 and 14 DPA in WT and Ts65Dn males. Graphs denote percentage of digits with closed wounds, and values above bars indicate the number of closed digits over total number of digits. (B) Representative sections of Mallory trichrome stained WT and Ts65Dn male digits. Dashed line represents the epidermis and the asterisk represents open wounds. (C) Number and percentage of digits with closed wounds at 7, 10 and 14 DPA in WT and Ts65Dn DS females. (D) Representative sections of Mallory trichrome stained WT and Ts65Dn female digits. *P* values indicate significance across all time points. Scale bars = 200 μm. Histological samples are shown with distal to the right, dorsal to the top.

In young Ts65Dn mice, wound closure is delayed only in males [35]. Given that aging is associated with delayed digit wound closure [42], we questioned if 6-month-old mice would also exhibit the wound closure anomalies observed in young Ts65Dn males. To investigate this, we harvested amputated digits at 7, 10, and 14 DPA from male and female 6-month-old Ts65Dn and WT mice (Fig. 7). Our previous findings in young male WT mice showed >50 % wound closure at 10 DPA and 100 % by 14 DPA, whereas only 20 % of digits in Ts65Dn young male mice had wound closure at 10 DPA and 30 % at 14 DPA [35]. However, unlike young Ts65Dn males, 6-month-old males showed no significant differences in the percentage of digits with closed wounds, with 0/12 closed digits at 7 DPA, 1/12 closed by 10 DPA, and over 50 % wound closure by 14 DPA (Fig. 7A, B; by Fisher’s exact group comparison test). Similarly, 6-month old females also showed no significant differences in percent digit wound closure on any day measured (Fig. 7C, D). Collectively, these findings support the conclusion that despite the negative effects on osteoclasts and osteoblasts in skeletally mature Ts65Dn mice, not all tissues in the P3 digit are negatively impacted, as epidermal closure that has slowed from 2 to 6 months of age in WT male mice is now similar to the consistently slow rate in Ts65Dn male mice at both ages.

## 4. Discussion

While the severity of bone anomalies are variable within the T21 population, there are skeletal characteristics that typify DS, including short stature [12,13], craniofacial alterations [66], short digits [67,68], early attainment of peak adult bone mass, low bone accrual and BMD [6,14], and a subsequent early onset of bone loss and steeper age-related decline in BMD [10,14]. Importantly, these skeletal features predispose the T21 population to fractures [15] across the lifespan and likely alter their capacity to repair bone; indeed, a recent report demonstrated disrupted fracture healing in an infant with T21 [16] that parallels our finding of impaired fracture healing in 16-week-old Dp16 DS mice [4]. Growing evidence suggests that fractures are more prevalent in the aging T21 population, especially in females after the age of 50 [11]. In this light, developing a deeper understanding of skeletal repair and regeneration across the lifespan in DS is a critical need. Here, using the P3 bone regeneration model in 6-month-old Ts65Dn mice, we demonstrate that P3 bone resorption and regeneration anomalies observed in young male mice [35] are exacerbated at 6 months of age, and that deficits in P3 bone resorption are observed in female mice.

The biphasic P3 regeneration cascade is characterized by an initial phase of osteoclast-mediated bone resorption [36]. The P3 resorption phase likely serves multiple functions: 1) ejection of the necrotic bone region, thus allowing a path for epidermal closure, 2) liberation of growth factors and cells within the extracellular matrix, and 3) integration of the remaining stump to the newly regenerated bone [46]. In young Ts65Dn male mice, P3 bone resorption is transiently attenuated, linked to reduced osteoclast recruitment and activity (measured by erosion perimeter), whereas young females showed no alterations [35]. By the age of 6 months, Ts65Dn males again showed reduced osteoclast recruitment and erosion perimeter, as well as a prolonged (yet not enhanced) resorption phase compared to WT males, demonstrating sustained osteoclast deficits initially found in young males. In 6 month-old Ts65Dn females, bone resorption is attenuated at 7 DPA, and while osteoclast recruitment (CTSK^+^ cells) is similar to that of WT females, osteoclast activity is reduced, demonstrating that Ts65Dn females acquire an osteoclast deficit as they mature. An important note here is that this deficit does not delay overall resorption, as both WT and Ts65Dn females have completed resorption by 10 DPA. Interestingly, aged (12-month-old) ICR female mice also exhibit unchanged P3 osteoclast recruitment but reduced osteoclast resorption; however, unlike 6-month-old Ts65Dn females, this resulted in delayed onset of and prolonged degradation [42]. Thus, due to the dissimilarities to the aged P3 degradation response, it is doubtful that the P3 osteoclast anomalies in Ts65Dn mice are driven only *via* accelerated aging. While the exact molecular underpinnings are still unknown, we provide evidence of temporally dysregulated control of osteoclastogenesis in 6 month-old Ts65Dn mice, characterized by decreased *Rank*, *Rankl*, and *Opg* expression at 7 DPA in both males and females. By 10 DPA, males and females diverge as Ts65Dn males exhibit enhanced *Rank* and *Rankl*, whereas females show enhanced *Opg*. It is likely that this favors prolonged degradation (yet not enhanced degradation, due to the reduced osteoclast number and/or activity) in males (Fig. 4) and the conclusion of degradation in females at 10 DPA (Fig. 3).

P3 bone regeneration is facilitated by blastemal-derived stem [69] and progenitor [36,47] cells that undergo proliferation and differentiation into bone *via* intramembranous ossification [38]. Young 8-week-old Ts65Dn mice present with sexually dimorphic P3 bone regeneration responses, with reduced bone regeneration observed in males and transiently enhanced bone regeneration observed in females [35]. By 6 months of age, both males and females demonstrate reduced osteoprogenitor differentiation and reduced blastemal proliferation, yet this only manifested as decreased bone regeneration in male mice. Ts65Dn females at 6 months of age are an intriguing case in that no differences were observed by μCT from 10 to 63 DPA, yet osteoprogenitors were transiently reduced at 7 DPA and gene expression for *Prx1*, *Runx2*, and *Osx* was reduced at both 7 and 10 DPA. We posit that despite reduced osteoprogenitors, Ts65Dn females may present with enhanced osteoblast activity to sustain complete P3 regeneration. Given that bone regeneration is comparable between WT and Ts65Dn females at 6 months of age, the fact that Ts65Dn females no longer show transiently enhanced regeneration points to a decline in their bone regeneration capacity, likely associated with a reduction in osteoprogenitor number despite the predicted enhanced activity. While further studies are needed to understand the regulation of osteoblast differentiation in Ts65Dn mice, evidence suggests that the altered P3 bone regeneration in skeletally mature Ts65Dn mice is not a direct recapitulation of typical aging, as aged ICR female mice show no defects in P3 osteoblast differentiation or blastemal proliferation [42]. Importantly, blastemal osteoprogenitor differentiation initiates prior to wound closure [48], whereas overt blastemal osteoid formation and bone mineralization initiate after wound closure [42], thus differences in wound closure can drastically alter bone regeneration timelines. In young Ts65Dn mice, P3 wound closure was delayed only in males [35], and notably, delayed wound closure is characteristic of the aged ICR P3 bone regeneration response [42]. Given that delayed wound closure would indeed delay P3 ossification, we predicted the bone regeneration defects observed in 6 month old Ts65Dn males were driven in part by delayed wound closure. Instead, the slow timing of epidermal closure in Ts65Dn mice at 2 months of age was maintained at 6 months of age in male and female Ts65Dn. Surprisingly, however, the timing and rate of wound closure in 6 month old WT mice was much slower and was similar to that of Ts65Dn mice. Collectively, these findings support the conclusion that a reduction in progenitor cell population and differentiation capacity, rather than alterations in wound closure, drive diminished bone regeneration in 6-month-old Ts65Dn male mice.

The blastema is the defining feature of epimorphic regeneration. P3 blastema formation is an early event that coincides with stump resorption and is initially localized to the periosteal and endosteal/marrow compartments [48]. At the culmination of stump resorption, the blastemal compartments merge concomitant with wound closure (Fig. 1). Blastema-derived osteoprogenitors differentiate and form new bone in a proximal to distal fashion to restore P3, and this response is associated with elevated expression of *Dlx5* [64], *Bmp2*, *Bmp4*, and *Bmp7* [37,65]. BMP signaling is required for blastema-derived osteogenesis [37], as exogenous Noggin inhibits P3 regeneration [65]. Importantly, BMP2, 4, and 7 are osteogenic during limb skeletogenesis [70], yet only BMP2 is required for adult bone homeostasis; the absence of post-natal limb BMP2 signaling results in spontaneous fractures, lack of periosteal activation, inhibition of periosteal callus formation and fracture bridging, and ultimately fracture non-union [71]. Consistent with reduced bone formation in Ts65Dn males, blastemal *Bmp2* was diminished whereas *Bmp7* expression is elevated compared to WT digits. These data, coupled with the minimal bone regeneration in the periosteal compartment (Fig. 3D), predicts the diminished *Bmp2* expression is closely associated with the attenuated bone regeneration in Ts65Dn males, despite the decreased *Noggin* expression. The periosteum is required for the overshoot in P3 bone volume and for the restoration of amputated P3 skeletal length [48], and indeed, Ts65Dn males showed both a diminished overshoot in bone volume and minimal bone regeneration in the periosteal compartment (Fig. 3B, D). While altered *Bmp* and *Dlx5* expression was observed in Ts65Dn females, they did not present with diminished *Bmp2* expression, consistent with no apparent differences in periosteal bone regeneration (Fig. 3H) and no differences in their capacity to regenerate P3.

Collectively, these studies demonstrate that skeletal maturation to 6-months-old in Ts65Dn mice negatively impacts osteoclast-mediated P3 bone resorption and intramembranous ossification, characterized by fundamental deficits in progenitor cell differentiation, cell activity, cell proliferation, and alterations in gene expression. As in young Ts65Dn mice, this response is sexually dimorphic, with males exhibiting more severe deficits than females. We provide *in vivo* evidence that male Ts65Dn mice exhibit less recruitment to both the osteoclast and osteoblast lineages. Conversely, Ts65Dn females show equivalent osteoclast recruitment yet less activity, and decreased osteoblast recruitment but equivalent bone regeneration, therefore suggestive of heightened osteogenic activity. We demonstrate that the DS trisomy in Ts65Dn does not impact all tissue types equally or necessarily have negative consequences on all tissues in DS, as epidermal wound closure was similar in Ts65Dn and WT. Importantly, these bone cell-specific deficiencies in bone regeneration in skeletally mature Ts65Dn mice have implications to the adult T21 population as the last several decades have seen substantial increases in the average life span of T21 individuals [72]. Similar to our findings of sexual dimorphism in the capacity for P3 bone regeneration in Ts65Dn mice, sexually dimorphic skeletal alterations have been observed in T21 individuals (reviewed [5]). For example, T21 individuals exhibit sexually dimorphic low BMD [3], and males show an earlier onset of developing low BMD compared to females [10]. T21 females, however, have shown a higher prevalence of fractures as they age compared to T21 men [11]. If the skeletal alterations during *de novo* P3 bone formation observed in skeletally mature Ts65Dn mice are recapitulated during bone healing in the adult T21 population, this could have profound consequences for this growing population.

## Supplementary Material

Supplementary Figures

Appendix A. Supplementary data

Supplementary data to this article can be found online at https://doi.org/10.1016/j.bone.2025.117648.

## Data Availability

The data that support the findings of this study are available from the corresponding author on reasonable request. The Down syndrome mouse models are commercially available from Jackson Labs.
