# Supplementary material for: Altered digit tip blastema differentiation and bone regeneration in skeletally mature Ts65Dn Down syndrome mice
Source: Bone. Author manuscript; Available in PMC 2025 Dec 10. (PMC12693721; doi:10.1016/j.bone.2025.117648)
Supplement: Supplementary Figures [file NIHMS2114191-supplement-Supplementary_Figures.pdf]

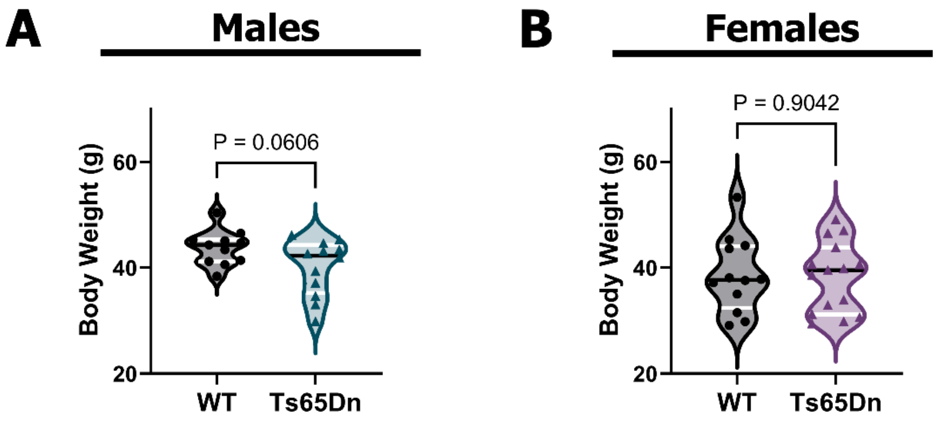

Supplementary Figure 1: Baseline body weight measurements of Ts65Dn and WT mice. No differences in body weight in males (A) or females (B) were observed in 6-month-old mice. Violin plots show individual data points, medians and 1st and 3rd quartiles, Student's t-test.

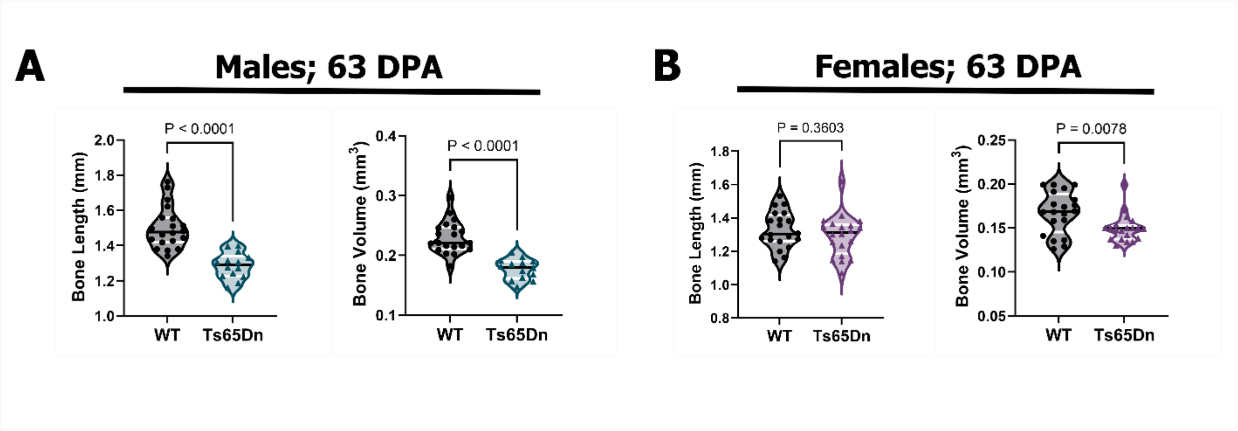

Supplementary Figure 2: Bone volume and length measurements at 63 DPA. (A) Bone volume and length at 63 DPA were significantly reduced in Ts65Dn males compared to WT control males. (B) At 63 DPA, WT and Ts65Dn females show no difference in bone length, whereas Ts65Dn females show reduced bone volume compared to WT females. Violin plots showing individual data points, medians and 1st and 3rd quartiles, Student's t-test.
